# Most of anti-glycolipid IgG-antibodies associated to neurological disorders occur without their IgM counterpart

**DOI:** 10.1186/s12929-019-0562-5

**Published:** 2019-09-06

**Authors:** Ricardo Dante Lardone, Fernando José Irazoqui, Gustavo Alejandro Nores

**Affiliations:** 1 Facultad de Ciencias Químicas. Departamento de Química Biológica Ranwel Caputto, Universidad Nacional de Córdoba, Ciudad Universitaria, X5000HUA Córdoba, Argentina; 2 Centro de Investigaciones en Química Biológica de Córdoba (CIQUIBIC), CONICET. Universidad Nacional de Córdoba, Córdoba, Argentina

**Keywords:** Glycolipid, Glycan, Anti-ganglioside IgG-antibodies, Autoimmunity, Neurological disorder, IgG/IgM discordance

## Abstract

**Background:**

Different neurological disorders frequently display antibodies against several self-glycans. Increasing evidence supports their pathogenic role; however, far less is known about their origin. Meanwhile, antibodies recognizing non-self glycans appear in normal human serum during immune response to bacteria.

**Methods:**

Using high performance thin layer chromatography-immunostaining, we comparatively evaluated humoral immune response (IgG and IgM immunoreactivity) against glycolipids carrying self-glycans (GM3/GM2/GM1/GD1a/GD1b/GD3/GT1b/GQ1b) and non-self glycans (Forssman/GA1/“A” blood group/Nt7) in sera from 383 patients with neurological disorders along with 87 healthy controls.

**Results:**

In contrast to no healthy controls having anti-self glycan IgG antibodies, one-fifth of patients’ sera had anti-self glycan IgG antibodies: remarkably, 60% of these occurred without IgM antibodies of the same specificity. Contrary to this unusual fact (anti-self glycan IgG occurrence without simultaneous presence of IgM having the same specificity ~ IgG/IgM discordance), all IgG antibodies against non-self glycans occurred simultaneously with their IgM antibody counterpart (i.e. 0% discordance). When analyzed closer, the IgG/IgM discordance frequency for anti-self glycans exhibited a dual trend: below 40% for IgG antibodies against GM2, GM1 and GD1b, and greater than 53% for IgG antibodies against the remaining self glycans. Interestingly, this discordance behavior was common to several different neurological disorders.

**Conclusions:**

Classic immunology principles indicate this anti-self glycan IgG/IgM discordance should not occur in an antibody response; its unusual presence is discussed within the “binding site drift hypothesis” context, where anti-self glycan IgG antibodies could originate from pre-existing IgG recognizing structurally-related non-self glycans.

**Electronic supplementary material:**

The online version of this article (10.1186/s12929-019-0562-5) contains supplementary material, which is available to authorized users.

## Background

Glycolipids are plasma membrane lipids displaying glycans as their hydrophilic head groups, which are accessible to binding by viruses, toxins and antibodies [1]. Anti-glycan antibodies are antibodies that, regardless of the immunogen that induces them, recognize saccharide sequences in one or more types of glycoconjugates [2]. Naturally occurring anti-glycan antibodies recognizing non-self carbohydrate sequences are routinely detected in normal subjects [3]. Typical examples are the ABO blood group agglutinins – i.e. sera from individuals of the blood group “0” contain antibodies that agglutinate blood group “A”/“B” red blood cells [4]. Since pioneering work of Springer [5] it is widely accepted that these antibodies are part of the normal immune response to bacteria colonizing respiratory or intestinal tract. A similar origin is described for IgM antibodies against a few self glycan-carrying glycolipids such as gangliosides GM1 and GD1b [6], although these normal antibodies are of low affinity and non-pathogenic [7]. In despite of this, immune reactivity recognizing self-glycolipids is often associated with autoimmune diseases [8]. In particular, a variety of neurological diseases present antibodies that recognize gangliosides (glycolipids abundantly found in nervous system) [9]. Unlike the large body of data indicating anti-ganglioside antibodies are responsible for triggering nervous system dysfunction through multiple mechanisms [10], much less is known about their origin. Infection of specific serotypes of *Campylobacter jejuni* cause Guillain-Barré syndrome associated with the presence of anti-GM1 antibodies [11]. These serotypes contain lipooligosaccharides carrying GM1-glycan (terminal tetrasaccharide) that can induce production of anti-glycan IgG-antibodies recognizing ganglioside GM1 (“molecular mimicry” hypothesis) [12]. Still, only a small minority of individuals infected with proper *C. jejuni* serotypes develops further neuropathy [13, 14], suggesting requirement for a “host susceptibility factor” that has not yet been identified [15]. On the other hand, it has been proposed that chronic neuropathy-associated anti-GM1 antibodies of the IgM isotype could originate by changes in the binding site of their normal counterpart (“binding site drift” hypothesis) [16]. “Binding site drift” can explain the “host susceptibility factor” of “molecular mimicry” hypothesis; therefore, both hypotheses can be regarded as complementary to explain the origin of anti-GM1 antibodies in disease [2]. Nevertheless, little is known about the origin of several other anti-self glycolipid antibodies associated to neurological diseases, especially for those having no “normal” IgM-antibodies [7].

In the present work, we comparatively evaluated the humoral immune response against various self and non-self glycan-carrying glycolipids in sera from patients with neurological disorders. We found remarkable differences between both antibody responses that were analyzed in the context of the “binding site drift” hypothesis, aiming for an explanation to the origin of disease-associated anti-glycan antibodies.

## Methods

### Human sera

Serum samples were obtained from 383 patients with early symptoms of neurological disorders: amyotrophic lateral sclerosis, *n* = 76; Guillain-Barré syndrome, *n* = 75; asymmetric motor neuropathy, *n* = 38; chronic inflammatory demyelinating polyneuropathy, *n* = 36; sensory neuropathy, *n* = 31; multifocal motor neuropathy, *n* = 25; sensory motor neuropathy, *n* = 23; Miller Fisher syndrome, *n* = 19; lower motor neuron disease, *n* = 18; mononeuropathy, *n* = 9; cranial neuropathy, *n* = 8; paraneoplastic syndrome, *n* = 6; multiple sclerosis, *n* = 4; diabetic neuropathy, n = 4; neuropathy with monoclonal gammopathy, n = 4; myasthenia gravis, *n* = 3; hereditary neuropathy, *n* = 2; amyotrophic neuralgia, *n* = 1; lumbosacral radiculitis, *n* = 1. These patients attended Neurology services from Hospital “Ramos Mejía” and Hospital Nacional de Clínicas “José de San Martín”, Buenos Aires, Argentina. Blood was collected before the patient underwent any immune treatment. After clot separation, sera were frozen and submitted to our laboratory for routine determination of anti-glycolipid antibodies. Normal human serum samples (*n* = 87) from healthy adult volunteers with negative serology for common infectious diseases were provided by Blood Bank of the University of Córdoba, Argentina. All procedures, performed in accordance with Ethical Guidelines on Research Involving Human Subjects [17] and with ethical standards as laid down in the 1964 Declaration of Helsinki and its later amendments, were approved by the Ethics Committee of CIQUIBIC-CONICET; informed consent was obtained from the patients.

### Glycolipids

The following biological materials were used as source of glycolipids: human brain for GM1, GD1a, GD1b, GT1b, and GQ1b; Sandhoff disease human brain for GM2; dog erythrocytes for GM3; chick brain for GD3; sheep erythrocytes for Forssman glycolipid (Forssman); human blood group “A” meconium for blood group “A” glycolipid; *Calliphora vicina* pupae for Nt7 glycolipid [18]. Folch upper phase of lipid extract [19] was purified by DEAE -chromatography [20] and HPLC on Iatrobeads silica-gel column [21]. Asialo-GM1 (GA1) was prepared by acid hydrolysis of cow brain gangliosides [22].

### High performance thin layer chromatography (HPTLC)-immunostaining

HPTLC with subsequent immunodetection (HPTLC-I) is considered the “golden standard” to detect anti-glycolipid antibodies and confirm autoreactivity results [23, 24]. Glycolipids (0.3 nmoles each) were separated on HPTLC plates (Merck) in the running solvent chloroform-methanol-aqueous 0.2% CaCl_2_ (45:45:10), using a tank designed to obtain highly reproducible chromatograms [25]. After air-drying, the plates were coated by dipping for 2 min in a 0.5% solution of poly (isobutylmethacrylate) (Aldrich Chemical Co., Milwaukee, WI, USA) in n-hexane-chloroform (9:1). Plates were blocked with BSA-PBSt (1% bovine serum albumin in phosphate buffered saline containing 0.05% Tween 20) for 1 h, incubated overnight with BSA-PBSt diluted serum, and washed thoroughly with PBSt. Binding was detected following 2 h incubation with BSA-PBSt diluted (1/1000) peroxidase-conjugated anti-human IgM (μ chain) or IgG (γ chain) goat antibodies (Sigma, USA). All the incubation steps were performed at 4 °C. After washing, color development was achieved in a substrate solution containing 2.8 mM 4-chloro-1-naphtol and 0.01% H_2_O_2_ in methanol-20 mM Tris-HCl buffer, pH 7.4 (1:29). The reaction was stopped after 20 min by washing the plates with PBSt. For usual immunostaining assay, sera were used at 1/20 dilutions. The presence of distinguishable immunostaining spot at these dilutions was considered a positive reactivity. In cases were only IgG was detected, higher serum dilutions were used (1/50, 1/500) to avoid potential IgM binding inhibition due to an IgG excess. To ensure data objectiveness, immunostaining results collected by first author (RDL) were checked against a blind assessment performed by one of the remaining authors (GAN).

### Statistical analyses

Antibody results were informed as categorical data and combined into groups for statistical purposes. Immunostaining against non-self glycans (GA1, Forssman, Nt7 and blood group “A” glycolipid for “0” and “B” blood group individuals) was grouped as “non-self glycan” reactivity. Since IgM populations against GM1, GD1b and GM2 have been described in normal human sera [7], reactivity against these glycolipids was considered a subgroup (“self glycan A”) within anti-self glycan antibody populations. Finally, response against GM3, GD3, GD1a, GT1b and GQ1b was counted as another subgroup (“self glycan B”). Data were examined by Chi-square or Fisher’s exact test with Prism 6 (GraphPad software, La Jolla, CA). Differences with *P* value < 0.05 were considered significant.

## Results

**Fig. 1:**
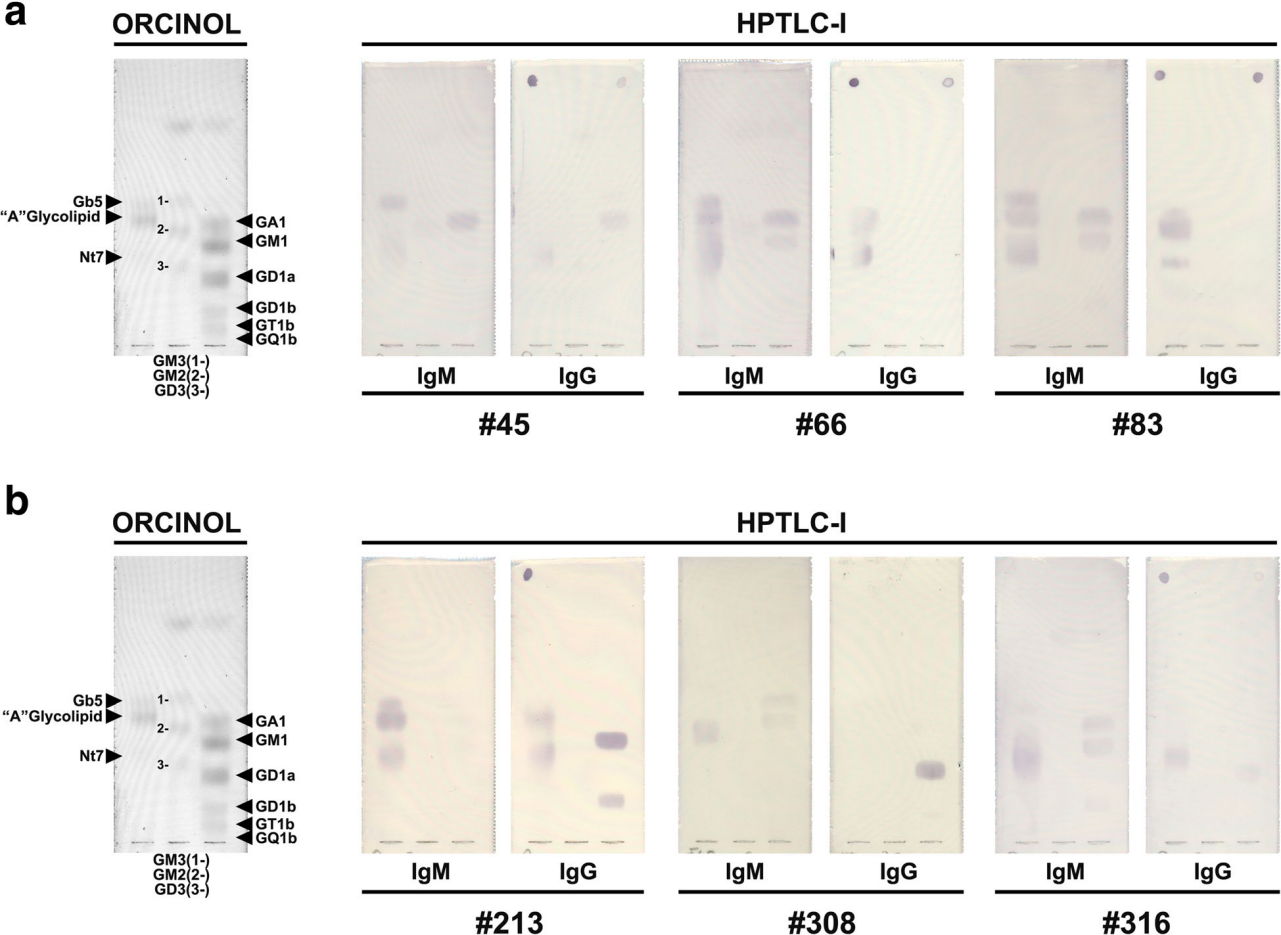
Reactivity of IgM- and IgG-antibodies against glycolipid self and non-self glycans. Representative results of the anti-non-self glycan and anti-self glycan immunoreactivity in (**a**) normal human sera and in (**b**) neurological disorder patients. On the left are glycolipids visualized using orcinol reagent

**Fig. 2:**
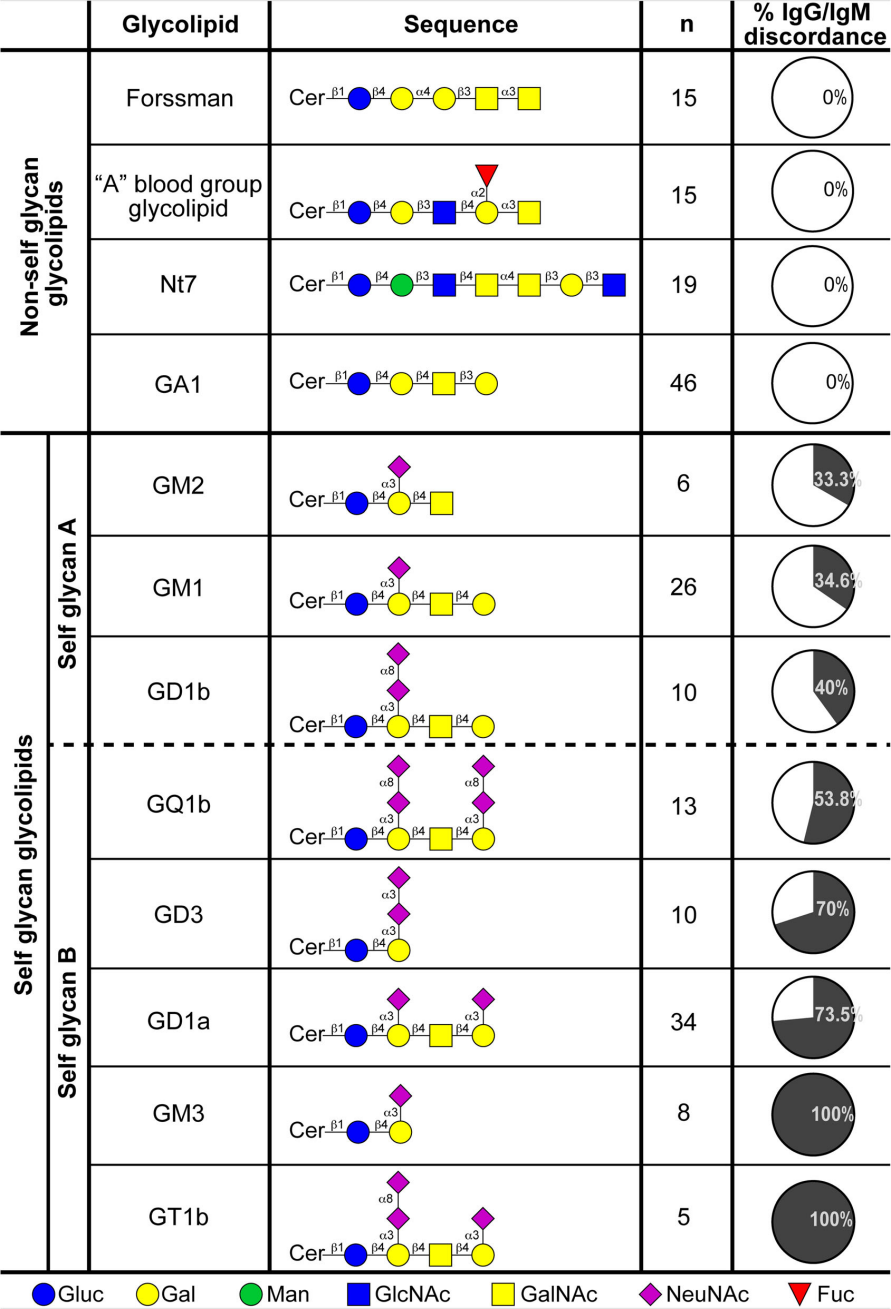
IgG/IgM discordance of IgG antibodies against diverse non-self and self glycan-carrying glycolipids analyzed in this study. Percentage of IgG/IgM discordance (percentage of samples having IgG antibodies with IgM antibodies of the same specificity) in patients with neurological disorders (positive for anti-self glycans and for anti-non-self glycans IgG antibodies of defined specificity). Oligosaccharide sequence of each glycosphingolipid recognized by antibodies is displayed. IgG/IgM discordance comparisons between “non-self glycan-”, “self glycan A” and “self glycan B” reactivities (these latter divided by a dashed line, see Methods) were all statistically significant (*p* < 0.0001, Fisher’s exact test). Anti-GA1 antibodies were measured in all patient samples, whereas the remaining anti-non self glycan antibodies were evaluated in a randomly selected fraction of patient sera (*n* = 30) that were positive for anti-self glycan IgG antibodies

**Fig. 3:**
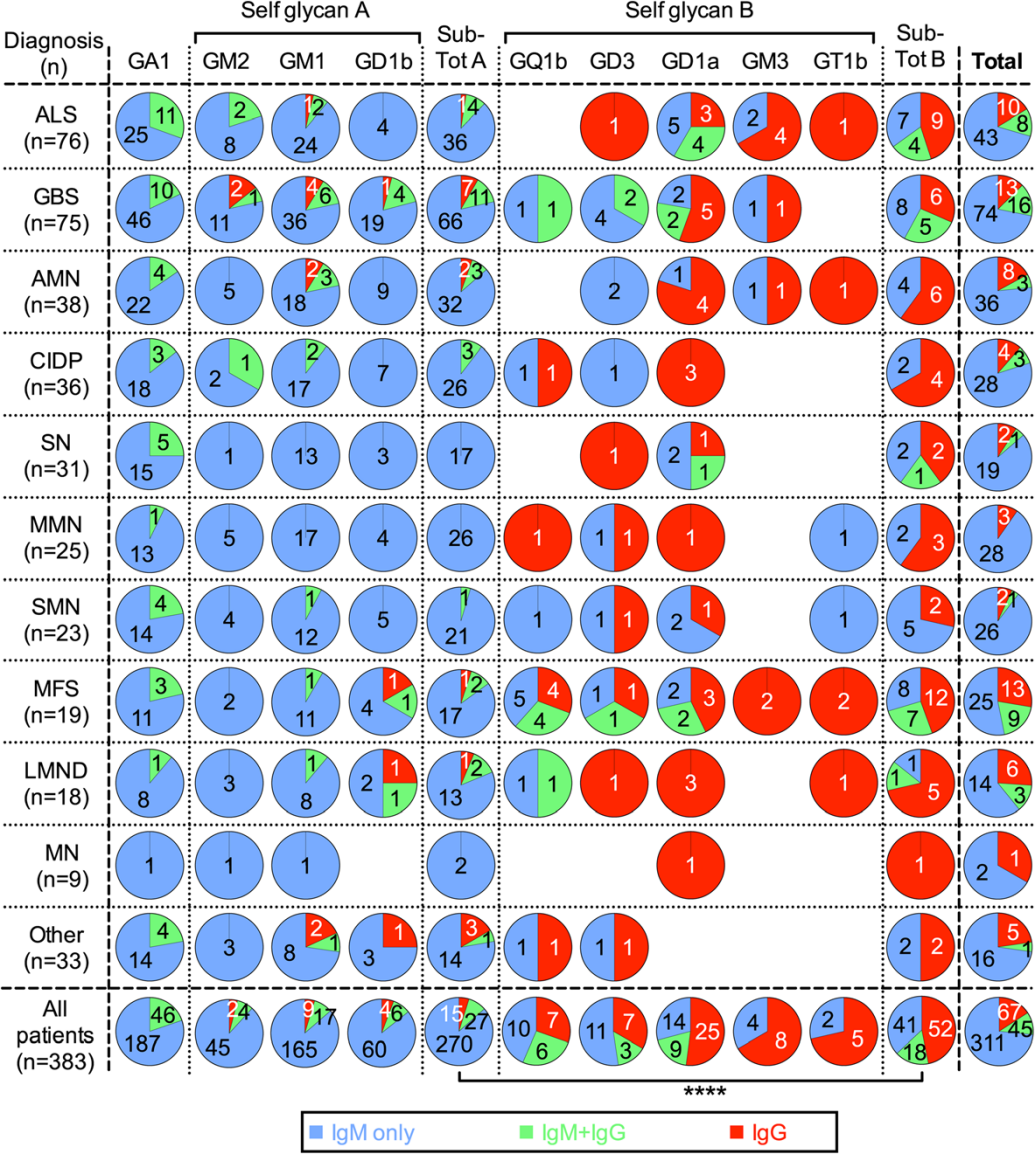
Distribution of anti-self glycan glycolipid and anti-GA1 antibodies in the different subpopulations of neurological disorder patients. Within each pie chart, the number of patients presenting antibody reactivity of the IgM (“IgM only”, blue), IgG (“IgG only”, red) or both isotypes (“IgM & IgG”, green) against the different self glycan glycolipids is displayed for each patient subpopulation and for all patients combined. The fraction of pie chart depicting “IgG only” reactivity represents the percentage of IgG/IgM discordance. Similar information is presented for anti-GA1 antibodies (non-self glycan glycolipid). “Self glycan A” subgroup comprises antibodies against GM1, GD1b and GM2, for which IgM reactivity populations have been characterized in normal human sera. “Self glycan B” subgroup includes antibody reactivity detecting the remaining glycolipids (GM3, GD3, GD1a, GT1b and GQ1b). “Sub-total A” plots sum the data for all “self glycan A” subgroup antibodies, while “sub-total B” column does it for all “self glycan B” subgroup antibodies. Far right column (“Total”) combines the data for all the anti-self glycan glycolipid antibodies. Total IgG/IgM discordance comparisons between each subpopulation of neurological disorder patients were statistically not significant. Comparisons of “sub-total A” versus “sub-total B” IgG/IgM discordance within each subpopulation of neurological disorder were not significant, whereas for “All patients” data combined, the comparison was statistically meaningful (Fisher’s exact test; ****, p < 0.0001). ALS, amyotrophic lateral sclerosis; GBS, Guillain-Barré syndrome; AMN, asymmetric motor neuropathy; CIDP, chronic inflammatory demyelinating polyneuropathy; SN, sensory neuropathy; MMN, multifocal motor neuropathy; SMN, sensory motor neuropathy; MFS, Miller Fisher syndrome; LMND, lower motor neuron disease; MN, mononeuropathy; Other, other neuropathies (see Methods and Additional file 1 for full details)

Previous reports have described the presence of anti-ganglioside antibodies in diverse diseases [26–33]. In a general screening searching for anti-glycolipid antibodies in neurological disorders, we analyzed serum samples from 383 patients, along with sera from 87 healthy controls. Using HPTLC immunostaining, we evaluated in both groups of samples the IgM and IgG antibody reactivity against self-glycan-carrying glycolipids: GM3, GM2, GM1, GD3, GD1a, GD1b, GT1b and GQ1b. For the immunostaining assay we used relatively low (1/20) serum dilutions, that allow detection of antibodies occurring at low titer. With variable immunostaining intensity, 75 patients (19.6%) were clearly positive for antibodies of the IgG isotype for at least one self-glycan antigen, while control sera were negative (Fig. 1). Some patients had IgG antibodies against two or more “self glycans”. Although similar percentages of antibody positive-patients have been published by several laboratories [26, 30, 34]), we observed a remarkable behavior of the antibody response: most of the IgG-reactivity against self glycans occurred without their IgM counterpart. Depending of the antibody specificity, the IgG/IgM discordance (IgG occurrence without simultaneous presence of IgM having the same specificity) ranged from 33% for those recognizing GM2 to 100% for antibodies recognizing GT1b (Figs. 2 and 3). Importantly, IgG/IgM discordance for the “self glycan A” subgroup (GM1, GD1b and GM2: all three reported to present IgM populations in normal human sera [7]) was significantly lower than that for the “self glycan B” subgroup (*p* < 0.0001; see Fig. 3, Additional file 1 and “Methods”).

The trend of IgG/IgM discordance for reactivity against self glycans was consistently observed across the different patient subpopulations, regardless of their diagnoses: evaluations within each patient subpopulation showed IgG/IgM discordances were always higher for the “self glycan B” subgroup. Statistical evaluation comparing discordance data between patient subpopulations having 20 or more individuals indicated the discordance magnitude was comparable throughout them (i.e. no significant differences; see Fig. 3). When compared individually within each patient subpopulation the discordance differences were marginally significant or not significant.

To assess if the observed IgG/IgM discordance was specific for anti-self glycan immune responses, we studied antibody reactivity to glycolipids bearing non-self glycans: Forssman, GA1, Nt7, and “A” blood group glycolipid, where the last one was considered non-self glycan for “0” and “B” blood group individuals (“A” individuals do not present anti-“A” antibodies). None of the patients exhibited IgG antibodies without their IgM counterpart, for any of the assayed non-self glycans (Figs. 1 and 2). As expected, the absence of IgG/IgM discordance for non-self glycans was significantly different compared to those from any of the anti-self glycan reactivity subgroups *(p <* 0.0001).

Examining co-occurrences of IgG populations against the different self glycans indicated a predominance (53 out of 75 patients, ~ 71%) of single self-glycan antigen reactivity (Additional file 2: Figure S1). Within the remaining 29% (22 patients), some isolated co-occurrences in antibody reactivity were observed for certain structures like GM1/GD1b (sharing terminal Galβ1-3GalNAc) or GD1b/GD3/GQ1b (b-series gangliosides), although no predominant events were detected (Additional file 2: Figure S1). Finally, regarding comparisons between GA1 and GM1 (structurally-related glycans), we found simultaneous occurrence with anti-GA1 IgG in 9 of 26 (34%) samples positive for anti-GM1 IgG antibodies.

For some randomly selected discordant serum samples, whole IgG fraction was removed using protein G-affinity columns. The non-adsorbed fraction (containing serum IgM-antibodies) had no anti-self glycan IgM reactivity (see examples in Additional file 3: Figure S2). These results indicate that absence of IgM reactivity in IgG-reactive sera is not due to IgG interference (antigen competition or anti-idiotype antibodies).

## Discussion

Humoral immune responses are complex and lead to different effector functions depending on their nature [35]. Glycan antigens have become important antibody targets in several medical contexts, such as vaccine design, diagnostic assays, and antibody-based therapies [36–38]. Immune response to non-self glycans is one of the early events in the defense against bacteria [39]. Natural infection of the gastrointestinal and respiratory tracts of the human body by pathogenic and non-pathogenic bacteria stimulate the immune system. Consequently, soon after birth, antibodies recognizing a variety of bacterial glycans are detected in children sera [6, 40, 41]. On the other hand, self-glycans carried by glycolipids have been associated to autoimmune diseases [9, 42]. The structural similarity of self and non-self glycans suggested both immune responses could be related [2]. In the present work, we characterized the antibody immune response against glycolipids carrying diverse self glycans in a large cohort of patients with neurological disorders, along with healthy controls. Antibodies of the IgG isotype recognizing self glycans were only detected in patient sera, with two types of results: some IgG-antibodies occurred with IgM of the same specificity, but most of them were discordant (i.e. without their IgM counterpart). To know if this discordance was exclusive of an autoimmune response, antibodies recognizing non-self glycan were studied in some discordant patient sera: in all cases where IgG reactivity was found, IgM of the same specificity was also present (Fig. 2), thus giving a relevant context to the IgG/IgM antibody discordance for anti-self glycan antibodies. Even though numerous studies and case reports in the literature have already portrayed antibodies of the IgG isotype against self glycan glycolipids in Guillain-Barré syndrome and related disorders, often they either provided information only for one or few glycolipids at the time (e.g. [27, 30]), used ELISA (instead of HPTLC-immunostaining) at higher serum dilutions than ours (e.g. [43, 44]) or, ultimately, did not include reactivity detection for glycolipids carrying closely-related, heteroantigenic glycans (e.g. [45, 46]). From a thorough revision of the literature we can say this discordance finding has not been remarked nor discussed before.

According to classical immunology principles, B-lymphocytes can be stimulated directly to produce IgM antibodies, whereas IgG production involves additional immune processes including T-cell cooperation. Thus, IgG antibody production should be accompanied by IgM antibodies and the IgG/IgM discordance would not occur. Since the IgG/IgM discordance was observed only in the antibody response directed to self glycans, its occurrence should be explained in the context of an autoimmune response. The occurrence of this antibody discordance does not seem to be related with a specific type of neurological disorder, since it was observed with similar magnitude in the different diseases evaluated. Previous work from our group showed that variations in antibody populations recognizing self-glycan glycolipids GM1 and GD1b are irrespective of motor neuropathy variants [47]. Some of these variations can even present certain heterogeneity among different patients suffering the same neuropathy [48, 49]. Each nerve of the peripheral nervous system has a specific function, and the experienced symptom/s in a neurological disease are determined by the type of nerve/s affected. There are various other factors that influence a neurological disease triggering: antibody affinity [50, 51], antigen density [52], membrane cholesterol content [53], sub-neuronal location of antigen [53], lipid environment [28], ceramide length [54], among others. The appearance of diverse antibody populations (as was also verified from the varied co-occurrences of IgG populations against the different self glycans) would constitute a random process [2] that, confluencing with the aforementioned factors could decide which nerve (or cell) will be targeted by an anti-self glycan autoimmune response. All this would be reflected as a lack of differences in IgG/IgM discordance rate between diseases. Overall, the presence of IgG/IgM discordance could represent a more general autoimmune phenomenon that merits further investigation.

**Fig. 4:**
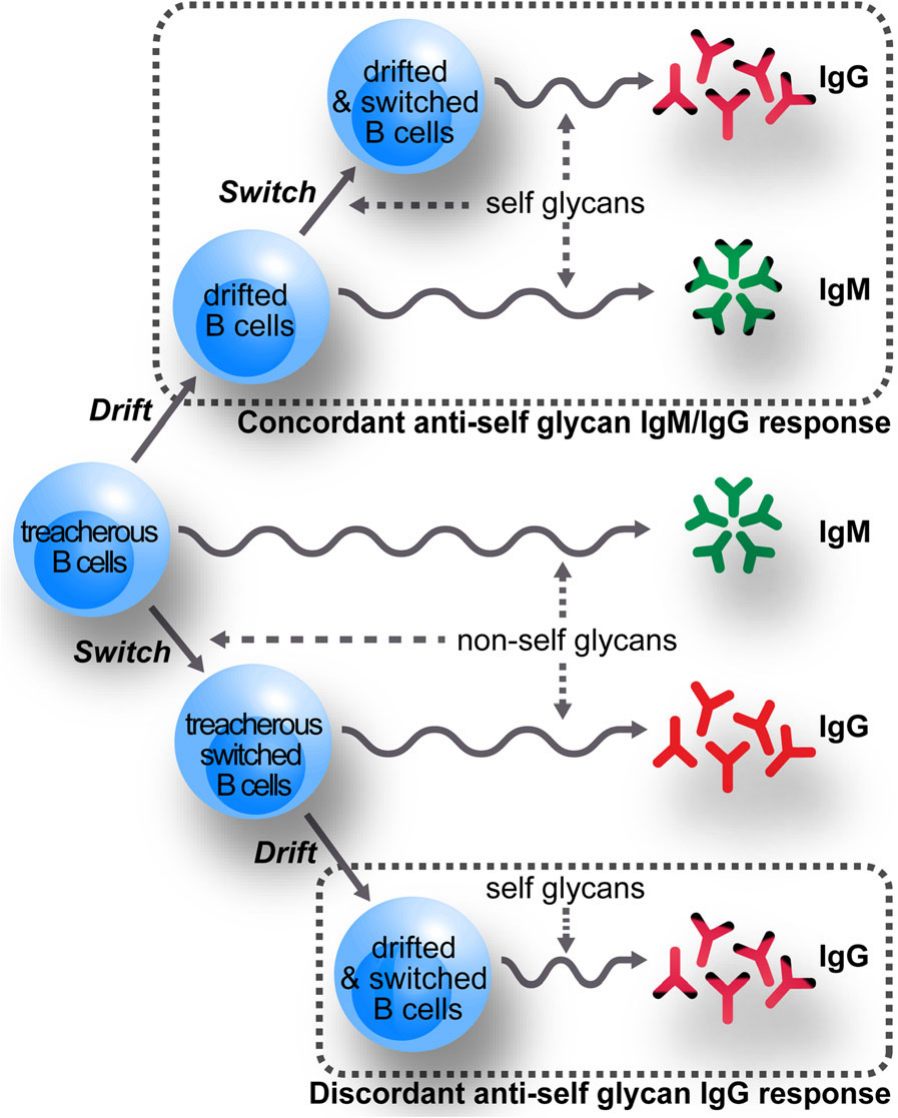
A hypothesis on the origin of antibodies against self glycans in patients with neurological disorders. Within the B cell repertoire able to respond against non-self glycans exist cell populations that recognize glycan molecules structurally related to self glycans. Although these so-called “treacherous” B cells cannot be stimulated by self glycans, during their activation by non-self structures they can undergo mutations that reshape the binding site, with some changes now leading to self glycans recognition (drift). These “drifted” B cells can then be activated by self glycans inducing the production of IgM antibodies (and IgG ones, if isotype switch occurs). Thus, these actions lead to a concordant anti-self glycan IgG/IgM antibody response. Alternatively, non-self glycan-stimulated “treacherous” B cell can switch their isotype to become “treacherous switched” B cells, producing anti-non-self glycan IgG antibodies. Subsequent drift events can now generate “drifted and switched” B cells that produce IgG after stimulation with self glycans. These latter steps can generate a discordant anti-self glycan IgG antibody response (i.e. without IgM antibodies)

Even though the “binding site drift” hypothesis was conceived to explain the origin of anti-GM1 antibodies in health and disease [2], it can be extended to other antibodies against self glycans. This hypothesis proposes that B-lymphocytes reactive to self-glycans originate from naturally-occurring B-lymphocytes recognizing structurally-related non-self glycans. In the B-cells repertoire involved in the immune response to non-self glycans, some cells (so called “treacherous”) can mutate its binding site in a way that now it can be activated by an endogenous or exogenous self glycan (Fig. 4). This process of specificity change in the B-cells was called “drift” because it would occur at random and, at least for anti-GM1 antibodies, it can follow different ways ending in antibodies having different fine specificities. If the “starting” B-cell undergoing “drift” is an IgM-producing cell, the resulting “drifted” B-cell can be stimulated to produce IgM antibodies or (if class switch is induced) an IgM/IgG response. Alternatively, as shown in Fig. 4, if the “drift” process acts on an already switched B-cell, the immune response will include only IgG-antibodies.

The discordance is less frequent in antibodies recognizing GM2, GM1 or GD1b (“self-glycan A”). This fact could be related to the presence in normal sera of IgM-antibodies recognizing these glycolipids [7]. Although they are low affinity antibodies that have no glycolipid-mediated biological activity [55], their occurrence is an indication for the normal presence of IgM-secreting B-cells that are candidate to be considered “treacherous” [2]. Autoimmunity triggering could arise from somatic mutations modifying the binding site of “treacherous” B cells and allowing high-affinity interaction with self glycans, resembling mechanisms observed for some carbohydrate-binding proteins (e.g. binding site point mutations leading to increased affinity of an anti-GA1 antibody Fab fragment [56] and an anti-blood group A [57]). This could allow a more frequent emergence of anti-self glycan, IgM-producing drifted B cells. From there on, some activated B cells could likely undergo isotype switching to contribute anti-“self glycan A” IgG antibodies. On the other hand, the large discordance incidence for the other self-glycan-carrying glycolipid antigens that do not have a naturally occurring IgM counterpart (“self glycan B”) implies that the “drift” process would occur more frequently at the IgG positive B-cells. In this case, potential binding site point mutations could modify antibody specificity along with affinity (reminiscing events like the generation of blood group B enzyme [58] or altered specificity of a lectin [59]), an expected result considering that somatic hypermutations levels of IgG are significantly higher than those of IgM [60].

## Conclusion

After exploring a large cohort of patients with different neurological diseases, our work revealed that antibodies of the IgG isotype against self glycans arose frequently without their corresponding IgM counterpart. In contrast, IgG antibodies against non-self glycans always exhibited their corresponding IgM. Interestingly, within the anti-self glycan IgG-antibody populations we found antigen-related, dual trend responses in this IgG/IgM discordance incidence (a low discordance frequency represented by GM2/GM1/GD1b, and a high discordance frequency encompassing GM3/GD3/GD1a/GT1b/GQ1b). Overall, these alternatives in IgG/IgM discordance behavior could result from B cells undergoing different paths during anti-glycan immune responses.

## Additional files

Additional file 1: Contingency tables for statistical analysis of the different comparisons. Tables were exported from the analyses performed in Graph Pad Prism 6. (XLS 59 kb)

Additional file 2: **Figure S1.** Co-occurrence in IgG antibody reactivity against different self glycans. Heatmap illustrating reactivity patterns for patient samples clearly positive for IgG antibodies against at least one self-glycan antigen. Columns denote each of the self-glycan antibodies, while rows represent the different patient samples. Positive reactivities are indicated in red; reactivity absence in gray. (TIF 3103 kb)

Additional file 3: **Figure S2.** Absence of anti-glycan IgM reactivity in IgG-reactive sera is not due to IgG interference. Randomly selected discordant serum samples were subjected to whole IgG fraction removal using protein G-affinity columns. Briefly, serum pH was adjusted by adding 1/10 volume of 1 M Tris buffer (pH 8). After filtration, the serum was passed through Sephadex columns with covalently bound Protein G (1 ml Protein G / 1 ml serum), with subsequent washes using 100 mM Tris buffer (pH 8.0). Examples for whole serum samples (“Before Protein G”) and non-adsorbed fractions (“After Protein G”) assayed for IgM and IgG using HPTLC-I are shown. (TIF 3814 kb)

## Data Availability

Data and materials are available from the corresponding author on reasonable request.

## Abbreviations

“A” blood group glycolipid: GalNAcα1-(Fucα1,2)3Galβ1-4GlcNAcβ1-3Galβ1-4Glcβ1-Cer
BSA-PBSt: 1% bovine serum albumin in PBSt
Forssman: GalNAcα1-3GalNAcβ1-3Galα1-4Galβ1-4Glcβ1-Cer
GA1: Galβ1-3GalNAcβ1-4Galβ1-4Glcβ1-Cer
GD1a: NeuNAcα2,3Galβ1-3GalNAcβ1-(NeuNAcα2,3)4Galβ1-4Glcβ1-Cer
GD1b: Galβ1-3GalNAcβ1-(NeuNAcα2,8NeuNAcα2,3)4Galβ1-4Glcβ1-Cer
GD3: NeuNAcα2-8NeuNAcα2-3Galβ1-4Glcβ1-Cer
GM1: Galβ1-3GalNAcβ1-(NeuNAcα2,3)4Galβ1-4Glcβ1-Cer
GM2: GalNAcβ1-(NeuNAcα2,3)4Galβ1-4Glcβ1-Cer
GM3: NeuNAcα2-3Galβ1-4Glcβ1-Cer
GQ1b: NeuNAcα2-8NeuNAcα2,3Galβ1-3GalNAcβ1-(NeuNAcα2,8NeuNAcα2,3)4Galβ1-4Glcβ1-Cer
GT1b: NeuNAcα2,3Galβ1-3GalNAcβ1-(NeuNAcα2,8NeuNAcα2,3)4Galβ1-4Glcβ1-Cer
HPTLC: High Performance Thin Layer Chromatography
Nt7: GlcNAcβ1-3Galβ1-3GalNAcα1-4GalNAcβ1-4GlcNAcβ1-3Manβ1-4Glcβ1-Cer
PBSt: phosphate buffered saline containing 0.05% Tween 20
