# Supplementary figures and images for: Most of anti-glycolipid IgG-antibodies associated to neurological disorders occur without their IgM counterpart
Source: J Biomed Sci. 2019 Sep 6;26:67. doi: 10.1186/s12929-019-0562-5 (PMC6729026; doi:10.1186/s12929-019-0562-5)

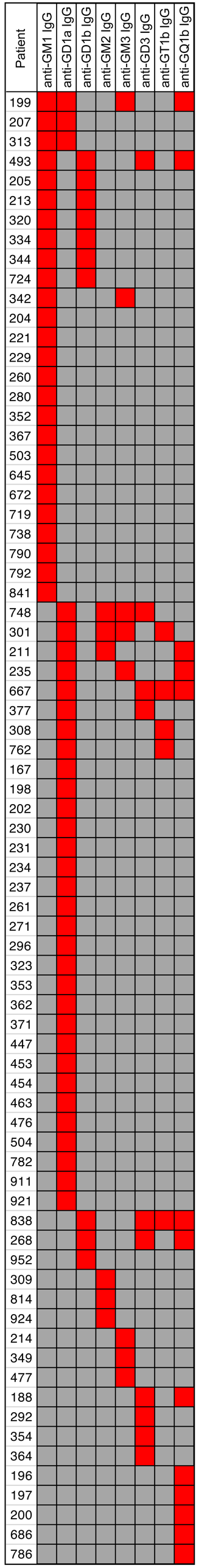

Supplement: Supplementary file 2 — Figure S1. Co-occurrence in IgG antibody reactivity against different self glycans. Heatmap illustrating reactivity patterns for patient samples clearly positive for IgG antibodies against at least one self-glycan antigen. Columns denote each of the self-glycan antibodies, while rows represent the different patient samples. Positive reactivities are indicated in red; reactivity absence in gray. (TIF 3103 kb) [file 12929_2019_562_MOESM2_ESM.tif]
